# Editorial: Early prevention of childhood obesity: identifying risks and implementing effective interventions

**DOI:** 10.3389/fendo.2026.1819834

**Published:** 2026-03-17

**Authors:** Valeria Calcaterra, Debora Porri, Gianvincenzo Zuccotti

**Affiliations:** 1 Pediatric and Adolescent Unit, Department of Internal Medicine, University of Pavia, Pavia, Italy; 2 Pediatric Department, Buzzi Children’s Hospital, Milan, Italy; 3 Department of Human Pathology in Adulthood and Childhood Gaetano Barresi, University of Messina, Messina, Italy; 4 Department of Biomedical and Clinical Science, University of Milan, Milan, Italy

**Keywords:** childhood obesity, children, complications, pediatrics, prevention

## 1 Introduction

Childhood obesity has become one of the most pressing global public health challenges of the 21st century. Its prevalence continues to rise worldwide, with profound consequences extending far beyond childhood into adult life, including type 2 diabetes, cardiovascular disease, and psychological and social complications.

Genetic susceptibility, intrauterine environment, early nutrition, lifestyle behaviors, and broader social and environmental determinants interact in complex and dynamic ways to shape long-term cardiometabolic health.

These interactions influence the development of adipose tissue, appetite regulation, insulin sensitivity, and energy balance, creating biological trajectories that may either promote resilience or increase vulnerability to obesity and its complications. Consequently, prevention strategies must move beyond late, symptom-oriented treatment and shift toward early identification of risk and timely, targeted interventions across the life course.

In recent years, the concept of prevention has evolved from a general, population-based approach to a more integrated and stratified model. Within this framework, primary prevention aims to reduce exposure to risk factors and promote healthy behaviors, secondary prevention focuses on the early identification of individuals at increased risk in order to implement timely and targeted interventions, and tertiary prevention seeks to limit complications and long-term consequences in those already affected. At the same time, scientific advances are paving the way for “precision prevention,” which takes into account individual susceptibility, early biomarkers, and critical windows of vulnerability to better tailor the timing and intensity of interventions.

Growing evidence shows that the prenatal period and early postnatal life represent particularly important windows of opportunity, since maternal health, early nutrition, and early-life exposures can leave lasting biological and metabolic effects. Nevertheless, childhood and adolescence remain crucial stages to consolidate or redirect these trajectories through family-, school-, and community-based interventions. Overall, effective prevention cannot rely on a single level of action, but requires coordinated strategies that integrate biological, clinical, behavioral, and social dimensions.

Despite growing knowledge, current prevention efforts remain fragmented, with limited integration between mechanistic research, clinical risk stratification, and real-world implementation. Closing these gaps is crucial to translate scientific advances into meaningful and sustainable reductions in the obesity burden. For this reason, the Research Topic *Early prevention of childhood obesity: identifying risks and implementing effective interventions* was designed to bring together complementary perspectives on early risk identification, biological mechanisms, vulnerable populations, and scalable prevention strategies.

To date, the Research Topic has received 9,294 topic views, 6,270 article views, and 1,295 article downloads, reflecting strong interest from the scientific community in early-life prevention. The accepted articles range from early biological programming to clinical risk stratification and population-level prevention, highlighting the translational value of integrated, life-course approaches to childhood obesity prevention.

## 2 Summary of articles

### 2.1 Prenatal programming and placental mechanisms

Spadafora et al. investigated the impact of maternal obesity on placental transcriptomic regulation of lipid metabolism, providing critical insights into the intrauterine origins of cardiometabolic risk. Using whole-genome correlation network analysis in term placentas, the authors identified specific mRNA and miRNA networks associated with cord blood free fatty acids and maternal leptin levels. Inflammatory pathways and the Rho signaling network emerged as key modulators of placental lipid handling, while several obesity-related miRNAs showed strong correlations with fetal lipid exposure. These findings reinforce the concept that the placenta is an active metabolic and epigenetic regulator shaping fetal programming and potentially predisposing offspring to future cardiometabolic disease, underscoring the importance of maternal metabolic health in early prevention strategies.

### 2.2 Clinical vulnerability and special pediatric populations

Sarli et al. examined obesity risk in children and adolescents with 22q11.2 deletion syndrome in a long-term, single-center cohort. While the prevalence of overweight did not differ significantly from the general pediatric population, obesity showed a distinctive bimodal age distribution, with peaks in early adolescence and late teenage years. Neuropsychiatric comorbidities and, most notably, the use of psychoactive medications emerged as major determinants of weight gain, with pharmacological treatment representing the only independent risk factor in multivariate analysis. These findings indicate that obesity in this population is largely modifiable rather than syndrome-intrinsic, highlighting the importance of anticipatory monitoring, multidisciplinary care, and cautious therapeutic strategies in vulnerable pediatric groups.

### 2.3 Biomarkers and precision prevention: the metabolomics perspective

Wan et al. employed an advanced metabolomics and machine learning framework to identify a robust obesity-associated metabolomic signature in children and adolescents. From over 900 detected serum metabolites, a 10-metabolite signature was derived that discriminated obesity with excellent accuracy. Higher signature scores were strongly associated with metabolic abnormalities, particularly hypertension and hypertriglyceridemia. The identified metabolites implicate pathways related to oxidative stress, protein catabolism, and glucocorticoid metabolism. This study provides compelling evidence for the potential of omics-based biomarkers in early risk stratification and precision prevention, supporting more individualized approaches to pediatric obesity and its metabolic complications.

### 2.4 Community and school-based prevention strategies

Zuccotti et al. described a large, integrated, school-based prevention initiative combining health education, physical activity promotion, and community engagement. The program involved more than 50,000 children aged 6–13 years and was structured around an educational phase followed by a final sporting event. High levels of participation and engagement demonstrated both feasibility and acceptability. By embedding healthy lifestyle promotion within a positive and inclusive value-based framework, this initiative illustrates how schools can function as powerful platforms for primary prevention, complementing biologically and clinically oriented strategies with scalable public health action.

### 2.5 Structured lifestyle management and continuous quality improvement

Lv et al. retrospectively evaluated the integration of a Plan–Do–Check–Act (PDCA) closed-loop management model into lifestyle intervention for children with simple obesity. Over six months, compared with conventional counseling, the PDCA approach achieved greater improvements in BMI-Z score, body fat percentage, waist circumference, insulin resistance, lipid profile, and key lifestyle behaviors, including physical activity, screen time, and unhealthy snack intake. Beyond weight-centered outcomes, this study shows how structured, iterative, and feedback-driven care pathways can sustain behavioral and metabolic change. These findings reinforce the need to move beyond episodic interventions toward longitudinal, adaptive, and implementation-oriented models of pediatric obesity management in real-world clinical practice and public health settings.

## 3 Emerging perspectives

Taken together, the contributions to this Research Topic underscore that effective prevention of childhood obesity must be multilevel, integrated, and initiated early in life. The evidence spans placental biology and fetal programming, clinical management of vulnerable pediatric populations, molecular biomarker discovery, population-based prevention strategies and structured, feedback-driven models for long-term lifestyle management that translate prevention principles into sustained clinical practice. A unifying message emerges: early-life windows represent unique opportunities for intervention, but their full potential can only be realized through coordinated approaches that bridge biological mechanisms, clinical practice, and public health implementation.

The convergence of omics technologies, developmental programming research, and implementation science further opens the path toward precision prevention, in which children at highest risk can be identified early and supported through tailored, timely, and context-sensitive interventions across family, healthcare, and educational settings.

## 4 Conclusions

This Research Topic reinforces the concept that childhood obesity is not simply a lifestyle condition emerging in later childhood, but the outcome of complex, lifelong trajectories that often originate before birth. By integrating mechanistic insights from placental biology, clinical evidence from high-risk populations, innovative biomarker approaches, and large-scale prevention initiatives, and structured implementation models for long-term lifestyle management, these contributions collectively advocate for a paradigm shift toward earlier, more personalized, and more comprehensive prevention strategies.

Future research should prioritize longitudinal, multi-omic, and implementation-focused studies to translate these insights into effective clinical practice and public health policy. Protecting metabolic health from the very beginning of life represents one of the most powerful and sustainable investments for improving population health across generations.

